# Impact of Axillary Lymph Node Dissection and Sentinel Lymph Node Biopsy on Upper Limb Morbidity in Breast Cancer Patients: A Systematic Review and Meta-Analysis

**DOI:** 10.1097/SLA.0000000000005671

**Published:** 2022-08-10

**Authors:** Nur Amalina Che Bakri, Richard M. Kwasnicki, Naairah Khan, Omar Ghandour, Alice Lee, Yasmin Grant, Aleksander Dawidziuk, Ara Darzi, Hutan Ashrafian, Daniel R. Leff

**Affiliations:** * Department of Surgery and Cancer, Imperial College London, London, UK; † Imperial College Healthcare NHS Trust, St. Mary’s Hospital, London, UK

**Keywords:** axillary lymph node dissection, axillary surgery, breast surgery, lymphedema, pain, quality-of-life, range of motion, sentinel lymph node biopsy, strength, upper limb morbidity

## Abstract

To evaluate the impact of axillary lymph node dissection (ALND) and sentinel lymph node biopsy (SLNB) on upper limb (UL) morbidity in breast cancer patients.

**Background:**

Axillary de-escalation is motivated by a desire to reduce harm of ALND. Understanding the impact of axillary surgery and disparities in operative procedures on postoperative arm morbidity would better direct resources to the point of need and cement the need for de-escalation strategies.

**Methods:**

Embase, MEDLINE, CINAHL, and PsychINFO were searched from 1990 until March 2020. Included studies were randomized-controlled and observational studies focusing on UL morbidities, in breast surgery patients. The study followed the Preferred Reporting Items for Systematic Reviews and Meta-Analyses (PRISMA) guidelines. The prevalence of UL morbidity comparing SLNB and ALND at <12 months, 12 to 24 months, and beyond 24 months were analyzed.

**Results:**

Sixty-seven studies were included. All studies reported a higher rate of lymphedema and pain after ALND compared with SLNB. The difference in lymphedema and pain prevalence between SLNB and ALND was 13.7% (95% confidence interval: 10.5–16.8, *P*<0.005) and 24.2% (95% confidence interval: 12.1–36.3, *P*<0.005), respectively. Pooled estimates for prevalence of reduced strength and range of motion after SLNB and ALND were 15.2% versus 30.9% and 17.1% versus 29.8%, respectively. Type of axillary surgery, greater body mass index, and radiotherapy were some of the predictors for UL morbidities.

**Conclusions:**

Prevalence of lymphedema after ALND was higher than previously estimated. ALND patients experienced greater rates of lymphedema, pain, reduced strength, and range of motion compared with SLNB. The findings support the continued drive to de-escalate axillary surgery.

Survival after breast cancer treatment has improved longitudinally as a result of more targeted and personalized treatments.^1^ This translates to many more breast cancer survivors experiencing long-term adverse consequences of therapy. Axillary treatment can leave patients with symptoms of arm and shoulder pain and/or reduced range of motion (ROM), with adverse impact on activities of daily living, health-related quality-of-life (HRQoL), and the ability to return to work.^1^ Moreover, axillary radiation and axillary lymph node dissection (ALND) are known to be significant risk factors for development of lymphedema and reduced ROM.^1^ While the introduction of sentinel lymph node biopsy (SLNB) in the 1990s reduced the number of ALND’s, there remains an important and yet to be quantified degree of arm morbidity associated with SLNB.^2^

Axillary de-escalation is driven by both a desire to minimize injury and a growing awareness of the oncological safety of axillary conservation.^3^ Understanding the effect of axillary surgery and differences in surgical intervention on postoperative arm and shoulder morbidity would allow for more effective resource allocation and would reinforce the importance of de-escalation strategies. Furthermore, it is critical to investigate which procedures or treatments are associated with the highest rates of morbidity so that clinicians can counsel patients regarding strategies with improved HRQoL outcomes.

The last systematic review investigating the adverse effects of breast cancer treatment on the upper limb (UL) was published in 2014,^1^ subsequently many important trials have been published.^3^–^6^ Whilst prior systematic reviews of the literature regarding UL morbidity have been performed, due to multiple methodologies of data presentation, the complexity of quantitative assessment through meta-analysis was precluded.^1^ Other systematic reviews focused on particular outcomes^7^,^8^ (eg, lymphedema or pain), medical therapies^9^ or included nonvalidated outcomes.^1^ Contemporary studies have improved in quality, including randomized-controlled trials (RCTs) and prospective cohort trials in the last decade, which might alter the results.^3^,^4^,^6^,^10^,^11^ In the current meta-analysis, only objective data measured by validated tools for lymphedema, ROM and strength or measured using validated questionnaires for UL function, pain, and HRQoL were included to ensure standardization wherever possible. We appraised data at an individual-study level with raw variables for preoperative to postoperative outcomes representing an internal difference metric which could then be expanded sequentially for study group synthesis. This was made possible by applying meta-analytical outcome weighting to each study and applying inverse variance methodologies. Data was synthesized from validated outcome measures to better characterize the adverse effects after breast cancer treatment on UL morbidity, in particular ALND and SLNB.

## METHODS

### Search Strategy and Selection Criteria

A systematic literature search was conducted using the databases of Embase, MEDLINE, CINAHL, and PsychINFO from 1990 to March 2020 according to the PRISMA (Preferred Reporting Items for Systematic Reviews and Meta-analyses) guidelines (Supplemental Digital Content 1, http://links.lww.com/SLA/E169). The review was registered with PROSPERO (CRD42020199311). The search terms included keywords as described in Supplemental Digital Content 2, http://links.lww.com/SLA/E170.

Included studies (Supplemental Digital Content 3, http://links.lww.com/SLA/E171) were RCTs and observational studies focusing on UL function in patients treated with breast surgery±additional therapies. Outcomes must be measured using validated tools. Papers that met inclusion criteria were reviewed using PRISMA methodological checklist (Supplemental Digital Content 1, http://links.lww.com/SLA/E169). Case reports, case series, letters, systematic and literature reviews, unpublished or ongoing trials, studies prior to 1990s, non-English studies, posttreatment interventional studies, and studies focusing only on chemotherapy/hormonal therapy were excluded. The articles were uploaded into COVIDENCE (www.covidence.org) which is a web-based screening and data extraction tool. Abstracts and titles were screened independently on COVIDENCE by 2 reviewers (N.A.C.B. and N.K.) and a third reviewer (A.L.) helped arbitrate in case of disputes. Full text screening was completed by NACB/NK and AL/OG.

### Data Extraction

Six outcomes were extracted based on the most common findings from reviewed studies (Supplemental Digital Content 4, http://links.lww.com/SLA/E172). Outcomes that were objectively measured for lymphedema, ROM and strength or measured using validated tools for UL function, pain, and HRQoL and studies focusing on ALND and SLNB were included in the review. Adverse effects of treatment were subcategorized based on time-point of data capture and defined as <12 months, 12 to 24 months, and more than 24 months. Methods of outcome reporting is described in Supplemental Digital Content 5, http://links.lww.com/SLA/E173.

### Risk of Bias Analysis

Two authors (N.A.C.B. and Y.G.) independently assessed the risk of bias of each study using the Risk of Bias 2 (RoB2) tool and Risk of Bias in Nonrandomized Studies of Interventions (ROBINS-I) tool for RCTs and nonrandomized studies, respectively. Disagreements were resolved by discussion, and when necessary, a third person arbitrated (A.D).

### Statistical Analyses

Meta-analysis was performed where possible on the prevalence rates of UL morbidity after ALND and SLNB. Pooled prevalence was calculated with a random effects model and presented with 95% confidence intervals (CI) utilizing the DerSimonian & Laird method as a between‐study variance estimator. Heterogeneity was assessed by the *I* ^2^ statistic (<30%—low, 31%–59%—moderate, >60%—high) and the Cochran *Q* statistic. Analyses were performed using Stata version 15 (Stata Corp LP; College Station, TX). Probability values (*P* value) ≤0.05 were considered statistically significant.

## RESULTS

**FIGURE 1:**
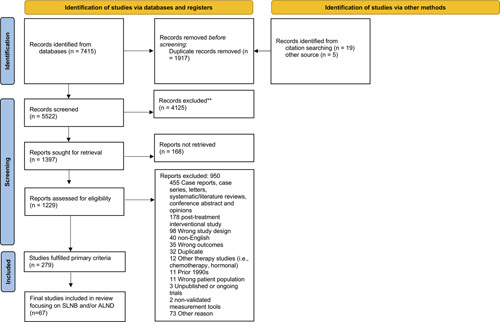
PRISMA flow diagram.

The initial search yielded 7415 articles (Fig. 1). Nineteen additional articles were identified from existing references and 5 additional articles were identified from other source. Two hundred and seventy-nine articles fulfilled our primary selection criteria. Articles were organized by outcome measure of interest and the 6 most common outcomes (Supplemental Digital Content 4, http://links.lww.com/SLA/E172) were selected, leaving 67 studies focusing on SLNB and/or ALND included in the review (Supplemental Digital Content 6, http://links.lww.com/SLA/E174).

### Lymphedema

#### Rates of Lymphedema After ALND and SLNB

**FIGURE 2:**
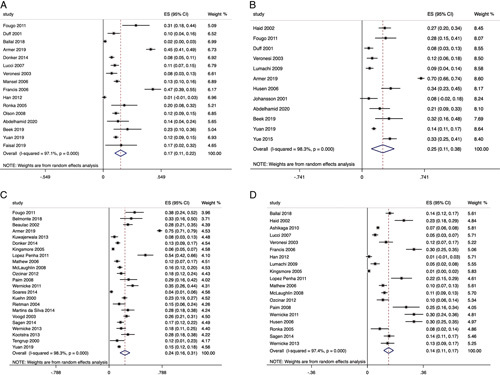
A, Prevalence of lymphedema after ALND at <12 months. B, Prevalence of lymphedema after ALND between 12 and 24 months. C, Prevalence of lymphedema after ALND at more than 24 months. D, Differences of lymphedema prevalence between ALND and SLNB.

Among 38 studies that reported the prevalence of lymphedema at different time intervals, the pooled estimate for the prevalence of lymphedema after ALND at <12 months (Fig. 2a),^3^,^4^,^6^,^11^–^23^ 12 to 24 months (Fig. 2b),^4^,^6^,^11^–^13^,^16^,^22^,^24^–^28^ and more than 24 months (Fig. 2c)^3^,^4^,^10^–^12^,^29^–^46^ was observed to be 16.5% (16 studies, n=3515, 95% CI: 11–22, *I* ^2^= 97.1%, *P*<0.0005), 24.6% (12 studies, n=1971; 95% CI: 11–38, *I* ^2^= 98.3%, *P*<0.0005) and 23.6% (23 studies, n=5288; 95% CI: 16.4–30.9, *I* ^2^= 98.3%, *P*<0.0005), respectively.

The equivalent pooled estimate for the prevalence of lymphedema after SLNB at <12 months,^14^,^15^,^18^–^20^,^47^,^48^ 12 to 24 months,^16^,^24^–^26^,^49^ and more than 24 months^31^–^36^,^43^,^44^,^50^ was observed to be 7.5% (6 studies, n=3866; 95% CI: 4.9–10.1, *I* ^2^= 79.8%, *P*<0.0005), 3.7% (4 studies, n=491; 95% CI: 1.8–5.6, *I* ^2^= 70%, *P*<0.0005), and 5.9% (11 studies, n=3136; 95% CI: 3.6–8.1, *I* ^2^= 82.8%, *P*<0.0005), respectively.

There was a statistically significant difference in the prevalence of lymphedema between ALND and SLNB, as illustrated in Fig. 2d (19 studies, n=9381; difference in pooled estimate=13.7%, 95% CI: 10.5–16.8, *I* ^2^= 97.4%, *P*<0.0005). All studies included in the meta-analysis^14^–^16^,^19^,^24^,^25^,^31^,^33^–^37^,^50^ reported higher rate of lymphedema following ALND.

#### Rates of Lymphedema After ALND and Axillary Reverse Mapping

The prevalence of lymphedema after ALND+axillary reverse mapping (ARM) at <12 months^6^,^11^,^22^,^23^ and more than 12 months^6^,^11^,^22^,^28^ was observed to be 3.3% (4 studies, n=657; 95% CI: 1.9–4.7, *I* ^2^= 0%, *P* =0.437) and 6.4% (4 studies, n=762; 95% CI: 1.9–10.9, *I* ^2^= 72%, *P* =0.013), respectively.

#### Immediate Versus Completion ALND

There was no significant difference in the rate of lymphedema when comparing immediate and completion ALND particularly in long-term follow-up. At 12 and 36 months, patients who underwent immediate ALND had no significant difference in lymphedema rate compared with completion ALND (17.8 vs. 8.6%, *P*=0.092^14^; 10.3% vs. 14.3%, *P*=0.65).^30^

#### ALND and Radiotherapy

ALND conferred a higher rate of lymphedema compared with axillary radiotherapy (RT) at 1 (8% vs. 6%, *P*=0.332), 3 (10% vs. 6%, *P*=0.08), and 5 (13% vs. 5%, *P*=0.0009) years.^3^ At 5 years, this difference was observed to be statistically significant (*P*=0.0009).^3^ Similarly, another RCT observed higher rates of lymphedema following ALND and breast irradiation (ALND-breast irradiation=22% vs. ALND=12%).^46^ Individuals who received ALND and regional lymph node radiation (RLNR) had the greatest 5-year risk of lymphedema (31.2%, Hazard ratio: 0.613, 95% CI: 0.403–0.935, *P*=0.023)^51^ when compared with ALND without RLNR (24.6%) and SLNB with RLNR (12.2%, Hazard ratio: 0.285, 95% CI: 0.149–0.545, *P*=0.0002). In a trial comparing RT versus non-RT, irradiated patients had a higher rate of lymphedema than nonirradiated (14% vs. 3%, *P*=not significant).^52^

#### Factors Increasing Risk of Lymphedema

Significant predictors for lymphedema included ALND (*P*<0.0001),^51^ chemotherapy (*P*<0.0001),^51^ high body mass index (*P*<0.0001),^51^ diabetes (*P*<0.05),^5^ palpable tumor (*P*<0.05),^5^ weight gain exceeding 10% of baseline value (*P*<0.001),^5^ and RLNR (*P*<0.0001).^51^ Lymphedema was also associated with a higher number of metastatic axillary lymph nodes (*P*<0.05),^53^ radical mastectomy (*P*<0.05),^53^ advanced age (*P*=0.006),^50^ the presence of seroma (*P*<0.001),^54^ and the amount of time passed after the procedure (*P*<0.05).^54^

### Pain

#### SLNB and ALND

**FIGURE 3:**
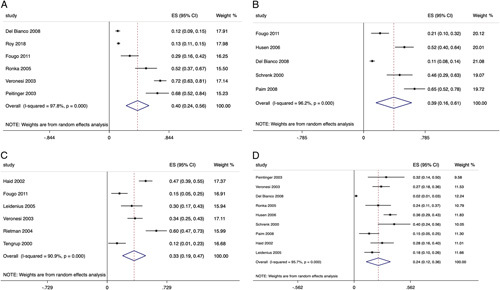
A, Prevalence of pain after ALND at <12 months. B, Prevalence of pain after ALND between 12 and 24 months. C, Prevalence of pain after ALND at more than 24 months. D, Differences of pain prevalence between SLNB and ALND.

According to 12 studies that reported the prevalence of pain after ALND at different time intervals, the pooled estimates at <12 months (Fig. 3a),^12^,^16^,^20^,^55^–^57^ between 12 and 24 months (Fig. 3b),^12^,^26^,^36^,^55^,^58^ and beyond 24 months (Fig. 3c)^12^,^16^,^24^,^40^,^46^,^59^ was 40% (6 studies, n=1335; 95% CI: 23.8–56.2, *I* ^2^= 97.8%, *P*<0.0005), 38.5% (5 studies, n=540; 95% CI: 15.7–61.4, *I* ^2^= 96.2%, *P*<0.0005), and 32.9% (6 studies, n=426; 95% CI: 18.8–47, *I* ^2^= 90.9%, *P*<0.0005), respectively.

In contrast, the pooled estimate for the prevalence of pain after SLNB at any different follow-up intervals was 21.7% (10 studies, n=1039; 95% CI: 13.9–29.5, *I* ^2^= 91.8%, *P*<0.0005).^16^,^20^,^24^,^26^,^36^,^55^,^57^–^59^ Nine studies comparing SLNB and ALND found the latter led to greater pain, with the pooled difference estimate of 24.2% (Fig. 3d) (9 studies, n=1788; 95% CI: 12.1–36.3, *I* ^2^= 95.7%, *P*<0.0005).^16^,^20^,^24^,^26^,^36^,^55^,^57^–^59^

### ROM

#### Prevalence of Reduced ROM and Flexion After ALND and SLNB

A greater proportion of patients experienced reduced ROM following ALND^6^,^14^,^21^,^22^,^26^,^27^,^44^,^46^,^58^,^60^,^61^ compared with SLNB,^14^,^26^,^44^,^47^,^48^,^60^ with a pooled estimate of 29.8% (11 studies, n=826, 95% CI: 17.5–42, *I* ^2^= 98.1%, *P*<0.0005) and 17.1% (6 studies, n=5809, 95% CI: 11.1–23.1, *I* ^2^= 96%, *P*<0.0005), respectively. Similarly, the pooled estimate for prevalence of reduced flexion after ALND^20^,^36^,^39^,^40^,^45^,^59^ and SLNB^20^,^36^,^48^,^59^ was 27.2% (6 studies, n=662, 95% CI: 15.2–39.2, *I* ^2^= 91.4%, *P*<0.0005) and 20% (4 studies, n=283, 95% CI: 11.5–28.6, *I* ^2^= 70.8%, *P*<0.0005), respectively.

#### Impact of Radiation on ROM

Reduced ROM was more prevalent in patients receiving axillary, supraclavicular, and/or chest wall RT compared with nonirradiated patients (52% vs. 15%, *P*<0.01).^52^ Similarly, a greater rate of reduced ROM was observed when combining ALND and axillary RT compared with ALND only (82% vs. 28%).^62^ In an RCT comparing ALND and axillary RT, ROM did not differ significantly between the 2 groups (1 years: *P*=0.29; 5 years: *P*=0.47).^3^

#### Predictors of Reduced ROM

ALND (*P*<0.001),^63^ RT (*P*<0.05),^63^ and side of dominance (*P*<0.05)^64^ were found to be significant predictors of decreased ROM. In addition, increased observation time (*P*<0.05),^53^ advanced age (*P*=0.011),^48^ radical modified mastectomy (*P*<0.05),^53^ higher body mass index (*P*<0.05),^53^ chemotherapy (*P*=0.002),^65^ presence of tumor-positive lymph nodes (*P*=0.014),^65^ expander-implant (*P*<0.05),^65^ and the latissimus dorsi flap (*P*<0.0001)^65^ were significant risk factors to decreased arm/shoulder function.

### Strength

#### Prevalence of Reduced Strength After SLNB and ALND

The pooled estimate of the prevalence of reduction in any strength (eg, grip, abduction, internal rotators strength) after ALND and SLNB was 30.9% (6 studies, n=825, 95% CI: 15.3–46.5, *I* ^2^= 96.5%, *P*<0.0005)^10^,^36^,^39^,^40^,^43^,^45^ and 15.2% (5 studies, n=437, 95% CI: 5.2–25.2, *I* ^2^= 91.8%, *P*<0.0005),^10^,^36^,^43^,^45^,^48^ respectively. The ALND group experienced more strength loss than the SLNB. In both SLNB and ALND, a decrease in strength of shoulder external rotation/abduction and ROM remained 5 to 7 years after surgery.^10^,^45^

#### Predictors and Association With Reduced Strength

Shoulder-arm function/strength 6 weeks following surgery and age were the greatest predictors of long-term shoulder-arm function/strength.^45^ Women who had surgery on their nondominant side demonstrated a larger loss of grip strength compared with those who had surgery on their dominant side (*P*=0.001).^66^ In addition, strength loss was associated with ALND surgery (*P*=0.046) and having received physical/occupational therapy (*P*=0.036) during follow-up.^60^

### UL Function

#### Prevalence of Reduced UL Function

The pooled estimate for the prevalence of reduced UL function after any breast cancer treatment was 34.4%% (3 studies, n=880, 95% CI: 17.2–51.7, *I* ^2^= 91.4%, *P*<0.0005).^48^,^64^,^67^

#### Association With Worse UL Function

Postmastectomy radiation and chemotherapy resulted in considerably worse (*P*=0.0093) UL function and HRQoL.^68^ Women with a history of diabetes (*P*=0.0249), rheumatoid arthritis (*P*<0.0001), or shoulder pain (*P*=0.002), a lower income (*P*<0.0001), and less health literacy (*P*=0.0062) were associated with poorer UL function.^68^

### HRQoL

#### HRQoL Post-SLNB and ALND

Studies comparing SLNB and ALND found that patients who received SLNB experienced better HRQoL than those who had ALND. Compared with global HRQoL scores preoperatively, the global scores postoperatively measured by European Organization for Research and Treatment of Cancer core quality-of-life (EORTC-QLQ-C30) improved by 15% (3 studies, n=155, 95% CI: 4.4–26.1, *I* ^2^= 99.9%, *P*<0.0005) in SLNB and 6% (3 studies, n=92, 95% CI: 0.7–11, *I* ^2^=99.8%, *P*<0.0005) in ALND.^57^,^69^,^70^

#### Association With HRQoL

Factors associated with poorer level of functioning and HRQoL were stage of disease (*P*=0.005),^71^ menopausal status (*P*=0.031),^71^ co-morbidities (*P*=0.032),^71^ chemotherapy (*P*=0.009),^71^ postoperative complications (*P*=0.02),^72^ upper-body function (*P*<0.001),^72^ greater psychological burden (*P*=0.001),^72^ had no confidante for social and emotional support (*P*<0.01),^72^ had unmet health care needs (*P*<0.001),^72^ and low health self-efficacy (*P*<0.001).^72^

### Risk of Bias Assessment

**FIGURE 4:**
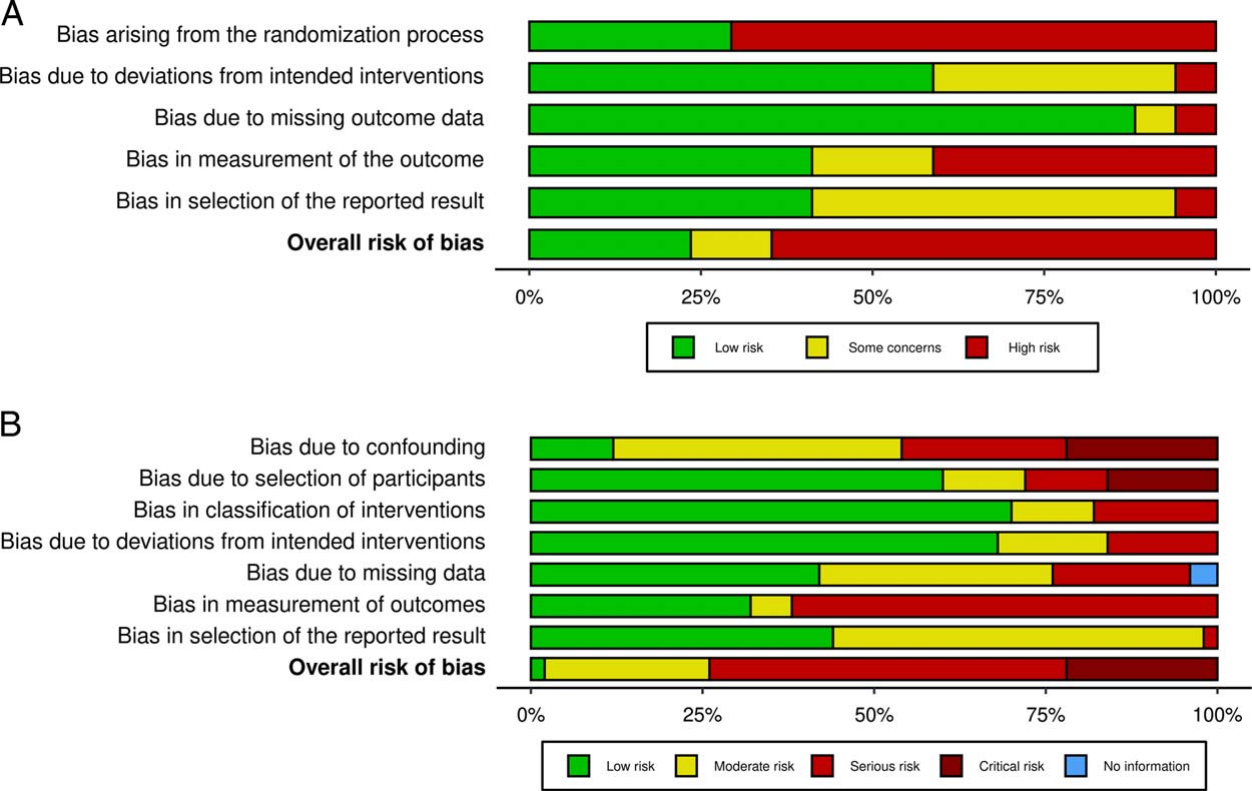
A, RoB2 tool for assessing risk of bias of randomized-controlled trials. B, ROBINS-I tool for assessing risk of bias of nonrandomized studies.

Overall, the risk of bias for included RCTs and nonrandomized studies were high or serious (Fig. 4). Risk of bias for individual studies can be found in Supplemental Digital Content 7, http://links.lww.com/SLA/E175 and 8, http://links.lww.com/SLA/E176.

## DISCUSSION

The pooled estimates for the short and long-term prevalence of lymphedema are higher than reported in prior systematic reviews.^1^,^7^ ALND was observed to have greater rates of lymphedema, pain, reduced strength, and ROM compared with SLNB. There is limited data available on UL function measured by DASH after ALND and SLNB, and there is no standardization in the measurement of UL morbidities making it difficult to compare between different treatments.

Prior systematic reviews focused on specific outcomes such as lymphedema^7^ or pain^8^ and medical treatments such as RT and surgery.^9^ One systematic review^1^ examined all the adverse effects related to breast cancer treatments, but did not perform a meta-analysis. As far as we know, this is the first systematic review where meta-analysis has been performed to better estimate the prevalence of lymphedema, pain, reduced ROM, and reduced strength following ALND and SLNB at different time intervals where possible.

The pooled estimates of the prevalence of lymphedema after ALND appear to increase with longer follow-up, which is consistent to findings from the review by Hidding *et al*, 1 although meta-analysis was not performed. In addition, the results are similar to the meta-analysis by DiSipio et al^7^ in which arm lymphedema increased longitudinally up to 2 years following diagnosis or surgery, at which point it appeared to decline. The authors also estimated 21% incidence of lymphedema after any breast cancer treatment.^7^ The prevalence of lymphedema after ALND in the current review using objective validated outcomes is higher compared with estimates by DiSipio et al,^7^ particularly in the long term (>24 mo, 23.6% vs. 18.6%).^7^ Compared with ALND, it is interesting that the prevalence of lymphedema following SLNB in our meta-analysis decreases with time. In this instance, time may not increase the prevalence of lymphedema; rather, the results may suggest that early lymphedema following SLNB is more prevalent, but it can resolve with time (with or without intervention). The pooled prevalence of pain appears to improve over time and this finding is consistent with the meta-analysis by Wang et al.^8^

In our current meta-analysis, there is a reduction of lymphedema rate when combining ALND and ARM approach compared with ALND only or SLNB in the first 12 months. Yuan et al,^11^ which was included in this meta-analysis, introduced a modified ARM approach called iDEntification and Preservation of ARm lymphaTic system (DEPART) that allows a thorough identification of the axillary lymphatic system and hence may further reduce the lymphedema occurrences. The ARM approach has the potential to minimize the rate of lymphedema, but it has not been adopted globally due to the uncertainty of its long-term oncological effects. The concern with the ARM approach is the anatomical crossover variations between breast and arm lymph nodes.^28^ Therefore, some of the preserved arm lymphatics may introduce the risk of metastasis, which can be a potential pitfall of this technique. Hence, large-scale studies with a longer follow-up period are required to assess the oncological safety of this method.

In comparison to SLNB, ALND patients have a higher rate of lymphedema, pain, decreased strength, and ROM. The current meta-analysis also demonstrates that the addition of axillary radiation to ALND increases the risk of UL morbidity especially lymphedema and reduced ROM which is consistent with a prior systematic review.^9^ While the type of axillary surgery is important in contributing toward the reduced ROM,^43^,^57^,^58^ arm positioning intraoperatively may also be a factor. The arm positioning for either SLNB or ALND is similar. Regardless of the type of breast cancer surgery they have, many breast cancer patients experience persistent arm/shoulder dysfunction for more than 12 months following surgery.^1^ Variations in shoulder ROM based on the type of surgery are not fully understood. When shoulder recovery patterns were analyzed by the number of lymph nodes removed, recovery patterns did not differ by the number of lymph nodes.^73^ This may suggest that arm positioning during the surgery may have an important role in determining the impact on ROM.

De-escalation of axillary surgery is motivated by a desire to reduce UL morbidities associated with ALND and subsequently improve patients’ HRQoL.^3^ The current meta-analysis adds to the evidence to de-escalate axillary surgery, such as SLNB, or to perform better surgery, such as exploring the ARM approach^6^ or the LYmphatic Microsurgical Preventive Healing Approach (LYMPHA)^74^ procedure when appropriate. There is also a need to provide patients and healthcare professionals with more information about the likely outcomes of their treatment. Therefore, resources can be allocated at an appropriate time to manage these life-debilitating morbidities.

There is a paucity of evidence on UL function following ALND and SLNB as indicated by DASH questionnaires. Although DASH was a frequently used patient-reported outcome for UL evaluation, there were few studies assessing UL function following ALND and SLNB. A recent RCT demonstrated that patients who are at increased risk of developing UL complications who received physiotherapy-led structured exercise had improved UL function as measured by DASH questionnaires 1 year following breast cancer treatment compared with usual care, which was proven to be cost-effective.^75^ The findings from this study support the implementation of an exercise program postbreast cancer treatment, strengthening the need to recognize patients who are at risk and intervene early.

The measurement of UL morbidities following breast cancer treatments is not standardized, making comparisons challenging. Many tools used are subjective, such as questionnaires, which may cause recall bias.^2^ Our group has found the use of wearable activity monitors helpful as a tool to assess function and UL activities after breast cancer treatment.^2^ This approach could improve the quality of data in the future when comparing the postoperative outcomes of limb function.

This study has several limitations that should be considered. Language bias might occur as we only included studies published in English. The lack of a universally agreed approach for documenting and assessing shoulder morbidity, making comparisons between treatment types and studies limited. The prevalence of lymphedema, reduced ROM and reduced strength may be under-reported as the outcomes measured by subjective tools were not included in the review. There was significant statistical heterogeneity in the studies included in the meta-analysis so that any interpretations of outcome measures require measured consideration. To mitigate these limitations, we included studies published from non-English speaking countries. Only objective data and data measured by validated tools were included in the meta-analysis to ensure standardization wherever possible. There is an urgent need to standardize the measurement of UL morbidities in future studies to reduce heterogeneity.

## CONCLUSION

This systematic review demonstrates that pooled estimates for the short-term and long-term prevalence of lymphedema after ALND are higher than previously estimated. ALND patients were observed to have higher rates of lymphedema, pain, reduced strength, and ROM compared with SLNB. There is an urgent need to develop a standardized tool with updated technology that is easy to use in a clinical setting to assess these morbidities to ensure better comparison between treatments and studies. Much work remains to be done to compare the effects of different breast cancer treatments and compute the costs associated with complications of treatment so that we can provide better support for breast cancer survivors. The findings reinforce the need to continue de-escalating surgical management of the axilla, including expanding the use of axillary irradiation instead of ALND in selected group of patients and exploring lymphatic microsurgery or ARM approach as feasible alternatives and adjuncts.

## Supplementary Material
