# Supplementary material for: Impact of Axillary Lymph Node Dissection and Sentinel Lymph Node Biopsy on Upper Limb Morbidity in Breast Cancer Patients: A Systematic Review and Meta-Analysis
Source: Ann Surg. 2022 Aug 10;277(4):572–80. doi: 10.1097/SLA.0000000000005671 (PMC9994843; doi:10.1097/SLA.0000000000005671)
Supplement: Supplementary file 2 [file sla-277-0572-s002.docx]

**Supplemental Table 2.** Search keywords

| Search | Keywords |
| --- | --- |
| Breast cancer | breast adj3 cancer* OR breast adj3 carcinoma* OR breast adj3 tumo?r* OR mamma* adj3 carcinoma* OR mamma* adj3 neoplasm* OR breast adj3 neoplasm* OR mamma* adj3 cancer* OR mamma* adj3 tumo?r* OR (subject heading) |
| Upper limb | upper limb* OR upper bod* OR (shoulder* or arm* or underarm* or elbow* or hand* or wrist*) OR upper extremit* OR (subject heading) |
| Function | (joint characteristics and functions) OR (movement (physiology)) OR (move* or function* or motion* or rotat* or strength* or disabilit* or impair* or dysfunction*) OR limit* OR (subject heading) |
| Surgery | surger* OR operate or operation* OR mastectom* OR lumpectom* OR reconstruct* OR (wide local excision* or wide excision* or WLE) OR breast resection* OR breast amput* OR (subject heading) |
| Axilla | (axilla* adj3 dissect*) OR axilla* OR (axilla* adj3 treat*) OR (axilla* adj3 clear*) OR (axilla* adj3 excis*) OR (underarm* or under arm* or armpit*) OR (lymph nod* adj3 excis*) OR (lymph nod* adj3 clear*) OR (lymph nod* adj3 resect*) OR (lymph nod* adj3 dissect*) OR (lymph nod* adj3 remov*) OR lymph nod* biops* OR (subject heading) |
| Radiotherapy | radiotherap* OR (external beam and (radiation or radiotherapy or irradiation)) OR (three dimensional and (radiation or radiotherapy or irradiation)) OR (3d and (radiation or radiotherapy or irradiation)) OR (three dimensional conformal radiation therapy) OR (whole breast and (radiation or radiotherapy or irradiation)) OR (cancer adjuvant therapy) OR (adjuvant chemoradiotherapy) OR (subject heading) |
