# Supplementary material for: Impact of Axillary Lymph Node Dissection and Sentinel Lymph Node Biopsy on Upper Limb Morbidity in Breast Cancer Patients: A Systematic Review and Meta-Analysis
Source: Ann Surg. 2022 Aug 10;277(4):572–80. doi: 10.1097/SLA.0000000000005671 (PMC9994843; doi:10.1097/SLA.0000000000005671)
Supplement: Supplementary file 3 [file sla-277-0572-s003.docx]

**Supplemental Table 3.** Inclusion and Exclusion Criteria

| Inclusion | Exclusion |
| --- | --- |
| Randomized-Controlled Trials (RCTs) and observational studies (cohort or case-control) are eligible. | Exclude case reports, case series, letters, systematic/literature reviews and opinion pieces, study protocol, in-vitro, animals or cadaveric studies, unpublished or ongoing trials |
| Studies assessing any upper limb functional outcome post breast and/or axillary surgery +/- additional therapies (radiotherapy, chemotherapy, hormonal therapy). | Exclude studies focusing on other therapies (i.e. chemotherapy, hormonal therapy) |
| Outcomes should be measured by validated tools | Exclude post-treatment interventional studies |
|  | Exclude non-English studies |
|  | Exclude studies prior to 1990s |
