# Supplementary material for: Impact of Axillary Lymph Node Dissection and Sentinel Lymph Node Biopsy on Upper Limb Morbidity in Breast Cancer Patients: A Systematic Review and Meta-Analysis
Source: Ann Surg. 2022 Aug 10;277(4):572–80. doi: 10.1097/SLA.0000000000005671 (PMC9994843; doi:10.1097/SLA.0000000000005671)
Supplement: Supplementary file 4 [file sla-277-0572-s004.docx]

**Supplemental Figure 4.** Six outcomes extracted based on the most common findings from reviewed studies

**
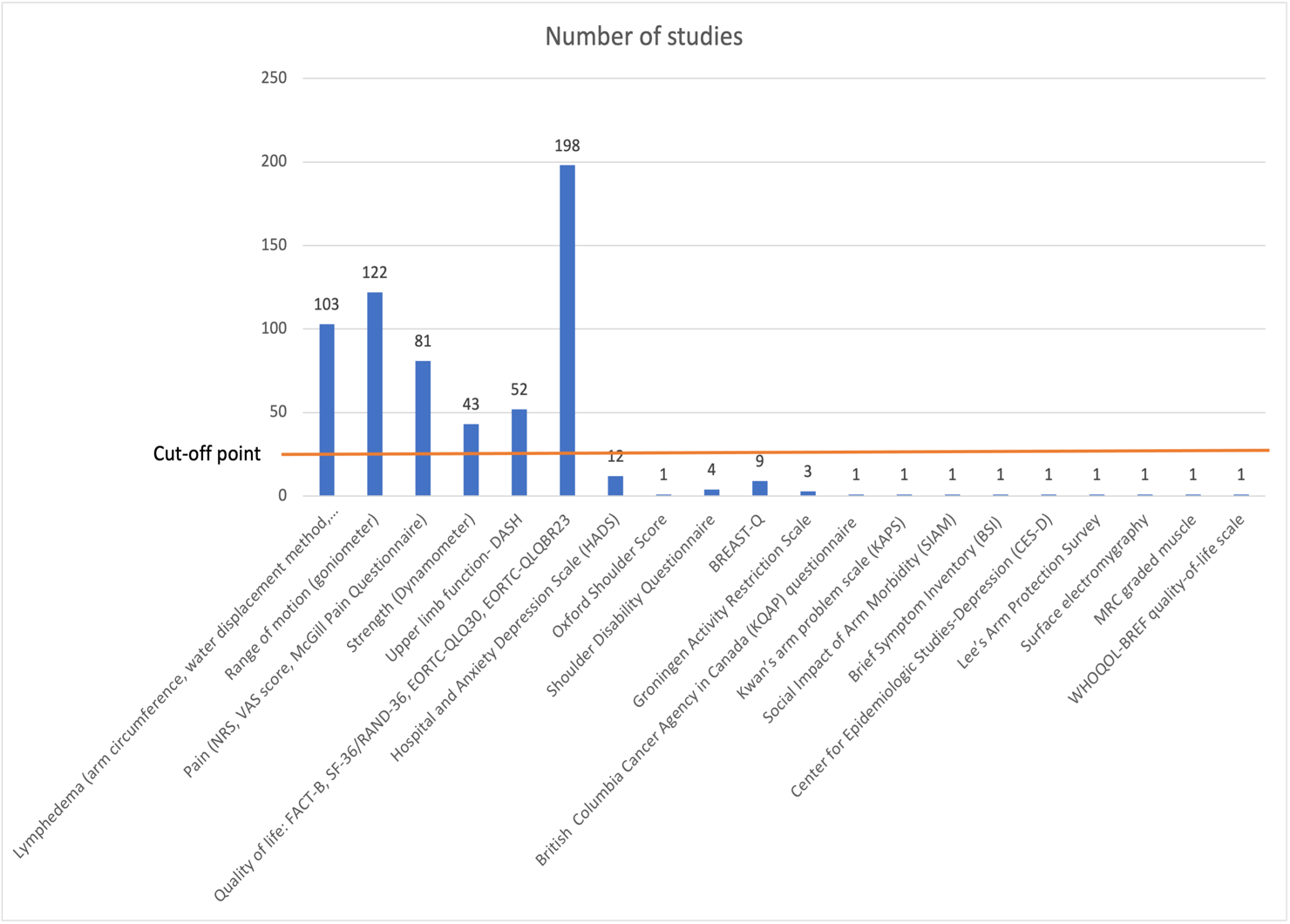
**

**Figure 4**. Outcomes measured in the literature. The six most-commonly reported outcomes were extracted. Cut-off point refers to >25 studies. Y-axis represents the number of studies with specific outcomes and x-axis represents the outcome reporting. (Numerical Rating Scale, NRS; Visual Analogue Scale, VAS; Disability of Arms, Shoulder and Hand, DASH; Functional Assessment of Cancer Therapy – Breast, FACT-B; 36-Item Short Form Survey, SF-36; European Organization for Research and Treatment of Cancer core quality of life, EORTC QLQ-C30; Breast cancer specific module, QLQ-BR23; Medical Research Council, MRC graded muscle; abbreviated World Health Organization Quality of Life, WHOQOL-Bref)
