# Supplementary material for: Impact of Axillary Lymph Node Dissection and Sentinel Lymph Node Biopsy on Upper Limb Morbidity in Breast Cancer Patients: A Systematic Review and Meta-Analysis
Source: Ann Surg. 2022 Aug 10;277(4):572–80. doi: 10.1097/SLA.0000000000005671 (PMC9994843; doi:10.1097/SLA.0000000000005671)
Supplement: Supplementary file 5 [file sla-277-0572-s005.docx]

**Supplemental Table 5.** Methods of Outcome Reporting

| Outcome | Measurement Tool and Definition |
| --- | --- |
| Lymphedema | Arm volume/circumference: More than 5%, >10%, or >200mL difference in volume (compared to baseline and/or contralateral limb) measured by water displacement method, tape measurement and perometry; or >2cm difference at any site on the upper extremity using tape circumferential measurements (compared to baseline and/or contralateral limb); Bioimpedance spectroscopy analysis: measurement increased by 10% or more compared to baseline or if impedance ratio is more than three standard deviations than normative data |
| Pain | Any pain (mild, moderate, severe, intermittent or persistent) measured by Visual Analogue Scale (VAS), numerical rating scale (NRS) or McGill Pain Questionnaire |
| Range of motion | Goniometer: Restriction in shoulder movement of more than 10 degrees, 15 degrees, 20 degrees or 25 degrees for each movement in comparison to contralateral arm or baseline measurement; or the limit for reduced mobility is set at 5% for flexion and abduction, and 10% for internal and external rotation as recommended by the American Academy of Orthopaedic Surgeons; or range of motion impairment less than 80° for rotation and less than 170° for abduction; or using a scale from 0 to 3 (0, no motion restriction; 1, minor restriction; 2, moderate restriction; and 3, severe restriction) |
| Strength | Dynamometer: Assessment of external rotators, internal rotators, abductors and adductors using a protocol described by Donatelli *et a*l or as recommended by The Americans Society of Hands Therapists |
| Upper limb function | Disability of Arms, Shoulder and Hand (DASH) questionnaire |
| Quality of life | Functional Assessment of Cancer Therapy - Breast (FACT-B), 36-Item Short Form Survey (SF-36), European Organization for Research and Treatment of Cancer core quality of life  (EORTC QLQ-C30) and breast cancer specific module  (QLQ-BR23) questionnaires |
