# Supplementary material for: Impact of Axillary Lymph Node Dissection and Sentinel Lymph Node Biopsy on Upper Limb Morbidity in Breast Cancer Patients: A Systematic Review and Meta-Analysis
Source: Ann Surg. 2022 Aug 10;277(4):572–80. doi: 10.1097/SLA.0000000000005671 (PMC9994843; doi:10.1097/SLA.0000000000005671)
Supplement: Supplementary file 6 [file sla-277-0572-s006.docx]

**Supplemental Table 6.** All studies reported the impact of SLNB and/or ALND on upper limb morbidities.

| Author | Design | Treatment/  Participants/Follow up | Measurement instruments in outcome | Main findings |
| --- | --- | --- | --- | --- |
| Abdelhamid 2020 | RCT | 98 (Group A: 49 ARM + positive preservation ALND vs Group B: 49 conventional ALND)  Follow up: Pre-surgery, 6 months, 12 months and 24 months | Lymphedema (arm circumference/volume)  ROM | - For group A: At 6 months, 12 months and 24 months post-op, the LR was 4.35%, 6.5% and 6.5% respectively - For group B: At 6 months, 12 months and 24 months post-op, the LR was 11.6%, 13.9% and 20.9% respectively - For group A: At 6 months, 12 months and 24 months post-op, the reduced ROM rate was 19.6%, 17.4% and 13.04% respectively - For group B: At 6 months, 12 months and 24 months post-op, the reduced ROM rate was 23.3%, 18.6% and 13.9.9% respectively |
| Arraras 2018 | Longitudinal cohort study | 138 (13 ALND, 82 SLNB, 28 no axillary surgery) + radiotherapy  Follow up: 3 years | EORTC QLQ-C30  EORTC QLQ-BR23 | - The ALND group scored lower (15-20 points) on the future perspective item than the other two axillary treatment groups at follow-up - No differences in pre- and post-treatment assessments across the different axillary treatment groups |
| Armer 2019 | Longitudinal prospective study | 488 (neoadjuvant chemotherapy followed by ALND)  Follow up: pre surgery, 1-2 weeks post-surgery and semi-annually for 36 months | Lymphedema (arm circumference/volume) | - LR at 3 years by ≥10% volume increase was 60.3% (95% CI 55.0–66.2%) and by ≥2 cm-circumference increase was 75.4% (95% CI 70.8–80.2%) - 3-year cumulative incidence of lymphedema and heaviness was 26.0% (95% CI 21.7–31.1%) and 30.9% (95% CI 26.3–36.3%), respectively |
| Ashikaga 2010 | RCT | 1975 ALND, 2008 SLNB  Follow up: baseline, 1 week, 2/3 weeks after surgery, every 6 months during the 3 year follow up | Lymphedema - volume  ROM- goniometer | - Shoulder abduction deficits ≥ 10% peaked at one week for the ALND (75%) and SLNB (41%) - Arm volume differences ≥ 10% at 36 months in ALND (14%) and SLNB (8%) - Numbness and tingling peaked at 6 months for the ALND (49%, 23%) and SLNB (15%, 10%) |
| Ballal 2018 | Retrospective review of prospective study | 745 (Group A – receiving SLNB only; Group B – primary ALND (with or without SLNB); and Group C – ALND as a second, separate procedure within 6 weeks of a positive SLNB)  Follow up:  6 months, 12 months post-surgery | Lymphedema - arm circumference  ROM-goniometer | - Overall LR: 8.2% at 12 months - No difference in LR between immediate or delayed ALND (17.8 vs 8.6%, p= 0.092) - Post-operative shoulder elevation, odds ratio (OR) = 0.390, 95% confidence interval (CI) = (0.218, 0.698) and abduction, OR = 0.437 (95% CI = (0.271, 0.705) reduced after ALND. No difference between immediate or delayed. |
| Beaulac 2002 | Retrospective Cohort study | 151 (ALND + Mastectomy or BCS)  Follow up: Mean 4.8 ± 0.2 years after surgery | Lymphedema -arm volume (water displacement)  ROM- goniometer  Strength- hydraulic hand dynamometer  FACT-B | - Total LR: 27.8% - Mastectomy or BCS patients had similar LR - Women with lymphedema in both surgical groups scored significantly lower on 4 of the 5 subsections than women without lymphedema, even after adjusting for other factors influencing HRQoL |
| Beek 2019 | RCT | 94 (46 standard ALND versus 48 ARM+ALND)  Follow up: pre-surgery, 6 months, 12 months and 24 months | Lymphedema -arm volume (water displacement) + arm circumference  ROM | - For standard ALND: At 6 months, 12 months and 24 months post-op, the LR was 21.4%, 23.4% and 32.3% respectively - For ARM+ALND: At 6 months, 12 months and 24 months post-op, the LR was 25%, 9.1% and 22.5% respectively - For standard ALND: 24 months post-op, the reduced ROM rate was 10% - For ARM+ALND: At 24 months post-op, the reduced ROM rate was 9% |
| Belmonte 2018 | Prospective cohort study | 68 SLNB 44 ALND  Follow up: at baseline (pre-surgery) and at the first and fifth year post-surgery. | Strength – dynamometer  ROM- goniometer  Lymphoedema – physician monitor  SF-36  FACT-B | - ALND group had significant loss of strength for IR (1.39 kg, p=0.001) and significant arm volume increase (132.45 mL, p=0.031) at 5 years. - ALND group had a greater number of patients with IR strength loss (38.7% vs. 13.6%, p=0.012) and a greater number of lymphedema requiring treatment (33.3% vs. 3.4%, p<0.001) than the SLNB group. - A loss of strength for shoulder ER, shoulder ROM, and HRQoL in Physical and Arm domains persisted at 5 years in both SLNB and ALND. |
| Bogusevisious 2013 | Prospective case-control study | 48 breast conserving surgery with SLNB vs ALND  Follow up: before surgery and after 1, 3, 6, 12, and 36 months | EORTC QLQ-C30 and QLQBR23 | - Emotional functioning and cognitive functioning were significantly better in the SLNB group than ALND group at the 12-month follow-up (86.7 vs 72.4, p<0.05) - SLNB scored sexual functioning significantly better at 3 (79.2 vs 53.1, p<0.05), 12 (80 vs 62.9, p<0.05), and 36-month (82.5 vs 73.8, p<0.05) follow-ups than ALND group. - The score on the arm symptom subscale was significantly worse at 1 (40.9 vs 19.1, p<0.05), 6 (30.6 vs 13.7, p<0.05), and 36- (31.7 vs 13.6, p<0.05) month follow-ups in the ALND than SLNB group. |
| Chrischilles 2019 | Cohort study | 833 breast cancer survivors (any treatment)  Follow up:  Mean follow up 22.1 (5.4) months after diagnosis | QuickDASH  FACT-B | - Patients who had post-mastectomy radiation and chemotherapy experienced significantly worse upper extremity dysfunction and HRQoL - Patients with lower income, lower health literacy and prior diabetes, arthritis or shoulder diagnoses had worse upper extremity disability |
| DeGroef 2016 | Longitudinal study | 100 sentinel node negative patients  Follow up: pre-surgery and 1 year post op | Lymphedema – arm circumference/volume  Pain (VAS)  ROM (Dr Ripstein Plurimeter-V gravity reference analogue inclinometer)  DASH  Strength (dynamometer) | - 8% of sentinel node-negative breast cancer patients had lymphedema - 50% of patients had pain, 30% had an impaired ROM, 8% had a decreased handgrip strength and 49% had an impaired shoulder function - Pain, shoulder range of motion, strength and shoulder dysfunctions changed significantly over one year (p<0.001). |
| Del Bianco 2008 | RCT | 341 patients randomized to the ALND group and 336 to the SLNB group  Follow up: every 6 months after surgery and yearly thereafter up to 24 months | Lymphedema – arm circumference/volume  Pain (NRS)  SF-36 | - 6 months after surgery, the SLNB group had significantly less lymphedema (OR 0.37, p= 0.005), movement restrictions (OR 0.47, p=0.005), pain (OR 0.52, p=0.006) and numbness (OR 0.65, p=0.016) with respect to the ALND group. - Lymphedema was also significantly reduced (OR 0.48, p=0.03) at 12 months and numbness (p<0.0001) remained significantly less frequent in the SLNB arm at all time points. |
| Devoogdt 2011 | Cohort study | 49 (mastectomy vs BCS + ALND)  Follow up: 3.4 years after surgery | ROM (goniometer)  Pain – VAS  Arm lymphedema – arm circumference | - At long term, 31% of the patients experienced impaired shoulder mobility, 18% developed lymphedema, 79% had pain and 51% mentioned impaired daily activities. - Between 3 months and 3.4 years after surgery, impaired shoulder mobility decreased from 57% to 31%. The incidence of lymphedema increased from 4% to 18%. Patients experienced an equal amount of pain but fewer problems with daily activities. - At 3.4 years, no significant differences between mastectomy and breast-conserving procedure were found. |
| Di Sipio 2009 | Cohort study | 316 (regional vs rural breast cancer survivors)  Follow up: 12 months | DASH  FACT-B | - In age-adjusted analyses, mean HRQoL scores of regional breast cancer survivors were comparable to their rural counterparts 12 months post-diagnosis (122.9, 95% CI: 119.8, 126.0 vs. 123.7, 95% CI: 119.7, 127.8; p>0.05). - Irrespective of residence, younger (<50 years) women reported lower HRQoL than older (50+ years) women (113.5, 95% CI: 109.3, 117.8 vs. 128.2, 95%CI: 125.1, 131.2; *p*<0.05) - Women who received chemotherapy, reported two complications post-surgery, had poorer upper-body function than most, reported more stress, reduced coping, who were socially isolated, had no confidante for social- emotional support, had unmet health care needs, and low health self-efficacy reported lower HRQoL scores. |
| Donker 2014 | RCT | 2402 ALND, 2404 axillary RT  Follow up: 1, 2 and 5 years | Lymphedema - arm circumference/volume | - Clinical sign of lymphedema in the ipsilateral arm was noted significantly more often after ALND than after axillary radiotherapy at 1 year (28% vs 15%, p<0.0001), 3 years (23% vs 14%, p=0.003), and 5 years (23% vs 11%, p<0.0001). - Arm circumference increase >10% of the ipsilateral upper or lower arm, or both was noted significantly more often after ALND than after axillary radiotherapy at 5 years (13% vs 5%, p=0.0009) |
| Duff 2001 | Cohort study | 100 (64 mastectomy, 36 BCS + axillary clearance)  Follow up: At least 1 year (pre-op, day 5, 6 months, 1 year, 2 years) | Lymphedema – arm volume  ROM- goniometer | - 10 % patients had significant arm swelling at 1 year. - Reduced arm movements were noted in the first week after operation but had returned to normal at 6 months. |
| Faisal 2019 | RCT | 48 (24 standard ALND – control group vs 24 ARM+ALND – study group)  Follow up: Pre-surgery and 6 months post-surgery | Lymphedema - arm circumference/volume | - LR in control group – 16.7% - LR in study group – 4.2% |
| Fougo 2011 | RCT | 106 (Group 1 – Assigned to axillary lymphadenectomy n=49. Group 2 – Assigned to observation n=57)  Follow up: 6, 12, 24 and 48 months after surgery | Lymphedema – arm circumference/volume  ROM | - The % of patients with morbidity and who had more than two complications was significantly higher in Group I - LR in group 1 was significantly higher than group 2 at 36 (38.3% vs 7%, p=0.001) months - Shoulder dysfunction in group 1 was significantly higher than group 2 at 36 (23.4% vs 3.6%, p=0.005) months |
| Francis 2006 | Cohort study | 155 (41 SLNB, 105 ALND, 9 no axillary surgery)  Follow up: 12 months after surgery | Lymphedema - arm circumference/volume | - LR at 12 months: total (68%), SLNB (17%), ALND (47%) |
| Golshan 2003 | Retrospective study | 125 (48 ALND, 77 SLNB) +/- chemotherapy +/- radiotherapy  Follow up: Minimum 1 year after surgery | Lymphedema – arm circumference/volume | - LR: ALND 27% and SLNB 2.6% |
| Haid 2002 | Cohort study | 140 had ALND, 57 had SLNB  Follow up: mean 18 (5–30) months | Lymphedema – arm circumference/volume | - No significant difference in HRQoL for both groups - Significant difference in overall complaints: (49% ALND group vs 19.3% SLNB group), pain (ALND: 47% vs SLNB: 19.3%), perceived limitation of motion (ALND: 43.5% vs. SLNB: 8.8%), numbness on the inner aspect of the affected arm (ALND: 49% vs SLNB: 0%), lymphedema (ALND: 27.1% vs SLNB: 3.5%), disability in activities of daily living (ALND: 50% vs SLNB: 12.3%) |
| Han 2012 | Cohort study | 97 (SLNB vs ALND)  Follow up: mean 9.6± 4.3 months (range, 3- 24 months) | Lymphedema – arm circumference | - 6 months: total (1%), SLNB (0%), ALND (1%) |
| Hojris 2000 | RCT | 84 (post mastectomy RT + systemic treatment vs systemic treatment alone)  Follow up:  median length of follow-up from mastectomy was 9 years (range 6–13 years) | Lymphedema – arm circumference/volume | - Lymphedema: 14% of the irradiated patients vs 3% of the non-irradiated patients (NS) - Slightly decreased shoulder morbidity in 45% of the irradiated women vs 15% of the non-irradiated patients, but moderate or more severe impairment was seen in only 5% of the irradiated patients and in none of the non-irradiated patients (p=0.004) - 17% of the irradiated patients and 2% of the non-irradiated patients found that impairment of shoulder movement caused symptoms (p=0.001) |
| Husen 2006 | Cohort study | 370 (Group A, SLNB only), (Group B, SLNB followed by a complete ALND during the first (and only) operation), (Group C, SLNB only in the first procedure, and a complete ALND in a second procedure)  Follow up: 23.5 months | Pain, lymphedema, numbness, reduced mobility, reduced strength, fatigue- interview questionnaire | - For the symptoms of pain (p=0.07), reduced mobility (p=0.9), reduced strength (p=0.66), and increased fatigue (p=0.52), there is no difference between the two ALND patient groups, although the frequency of these symptoms is considerably higher in both groups compared to the SLNB alone group (p<0.0001). - Two-step axillary clearance represented the most important influential factor of the development of arm morbidity symptoms of swelling and numbness. - Other factors that had an influence on the development of arm morbidity were: tumour location in upper lateral quadrant, age <55, chemotherapy and BMI>25. |
| Jassim 2013 | Cross-sectional | 239 (any treatment)  Follow up: early after diagnosis (≤1 year since diagnosis), transitional period (>1 and ≤5years since diagnosis) and long-term survivors (>5 to ≤10 years). | EORTC QLQ-C30 and QLQBR23 | - Mean score for global health of 63.9 (95% CI 61.21-66.66). - Among functional scales, social functioning scored the highest (Mean 77.5 [95% CI 73.65-81.38]) whereas emotional functioning scored the lowest (63.4 [95% CI 59.12-67.71]). - The most distressing symptom on the symptom scales was fatigability (Mean 35.2 [95% CI 31.38-39.18]). |
| Johansson 2001 | Cohort study | 90 women undergoing axillary dissection combined with mastectomy or segmental resection  Follow up: pre-op and monthly for 6 months with 1 and 2 year follow ups | Lymphedema - volume (water displacement method)  ROM (goniometer)  Strength (dynamometer) | - There were significant differences in lymphedema between the ART and NRT groups at the 1- and 2-year follow-ups (p<0.05) and (p<0.02), respectively. - At 1 and 2 months, there were no differences between the groups at any ROM. At 3 months, the ART group had reduced mobility in abduction (p=0.003) and flexion (p=0.017), and at 6 months, they had reduced mobility in external rotation (p=0.021). - At 6 months, 1 year, and 2 years, isometric muscle strength in the flexors, adductors, and internal rotators of the shoulder decreased significantly in the entire group. |
| Kingsmore 2005 | Retrospective cohort study | 2122 women treated for invasive breast cancer  Follow up: Median 8 years since diagnosis | Lymphedema – Objective persistent arm swelling noted by clinician | - LR: SLNB (20/146, 5%); ALND (69/1099, 6%) - LR: Axillary radiotherapy only (4/92, 4%) - Addition axillary radiotherapy to SLNB and ALND increased the LR: SLNB + ART (26/230, 11%), ALND + ART (17/123, 14%) p<0.001) |
| Koehler 2018 | Longitudinal prospective cohort study | 36 (AWS vs non AWS)  Follow up:  at 2, 4, and 12 weeks and 18 months following breast cancer surgery | Lymphedema – arm circumference/volume, bioimpedance spectroscopy (BIS), and trunk tissue dielectric constant measurements  ROM (goniometer)  Pain (VAS)  DASH | - No statistical group differences between the AWS and non-AWS groups related to upper extremity percent volume difference (F=.07, p = 0.79) - The AWS group had statistically impaired abduction AROM (F=4.5, p=0.005) and PROM (F=3.6, p=0.02) at 2 and 4 weeks but not at subsequent visits - Shoulder flexion AROM was statistically lower in the AWS group compared to the non-AWS group at 2 weeks (p<0.05) but not subsequent visits - Both AWS and non-AWS groups had a decrease in DASH scores from 2 weeks to 12 weeks, but at 18 months the AWS group had an increase in DASH scores while the non-AWS group had a continued decrease in DASH scores |
| Kootstra 2013 | Longitudinal cohort study | 110 (SLNB vs ALND)  Follow up: pre-surgery (T0) and 6 weeks after surgery (T1), 7 years later (T2) | ROM (goniometer)  Strength (dynamometer)  Lymphedema- arm circumference | - ROM (excluding external rotation), abduction strength, and arm volume were all reduced 7 years after surgery compared to pre-surgery levels - Those with ALND demonstrated 70% greater forward flexion disability, increased arm volume, and clinically relevant impairments than women with SLNB (41%) - The strongest predictors of long-term shoulder–arm function are shoulder–arm function at 6 weeks following surgery and age |
| Kopec 2013 | RCT | 744 (SLNB vs ALND). Women who had sentinel node–negative breast cancer were enrolled in the HRQoL component of NSABP B-32  Follow up: 6 months after surgery | ROM (goniometer)  Lymphedema - volume (water displacement method)  DASH survey | - 1/3 experienced reduced ROM - 1/3 avoided the use of the arm 6 months after surgery - Limitations in work and other regular activities were reported by 1/4 - In multivariable analyses with a global HRQoL score as a continuous variable, significant predictors of HRQoL were ROM (RC=0.017 per degree; p<0.001) and neuropathy (RC=−0.715; p<0.001) |
| Kuehn 2000 | Cohort study | 396 (breast conserving therapy or mastectomy including axillary dissection)  Follow up: mean 34 months (median 32, range 6–96) after surgery | Lymphedema – arm circumference/volume  ROM (goniometer)  Strength – five-point score | - Total LR: 22.7% - 115 patients (29.0%) declared ‘arm symptoms’ as their most important problem - Mean restriction of 21◦ for abduction and 12◦ for forward flexion compared to the contralateral arm are statistically significant (Wilcoxon test, p<0.001). - Restriction of isometric strength of at least one score point was observed in 172 (43.4%) of patients - 24 patients (56.6%) had no measurable reduction of arm strength compared to the contralateral arm |
| Kuwajerwala 2013 | RCT | 199 (SLNB vs SLNB+ immediate ALND or completion ALND)  Follow up: every 6 months up to 24-36 months | Lymphedema – arm volume | - LR for node-positive SLNB+ALND was 10.3% and node-negative SLNB+ ALND was 6.8% - Comparison between immediate and completion ALND lymphedema was not statistically significant (10.3% vs. 14.3%, p= 0.65). |
| Leidenius 2005 | Cohort study | 92 SLNB only or 47 ALND  Follow up: 3 years post op | Lymphedema – arm circumference/volume  ROM (goniometer)  Pain (VAS) | - Breast pain in SLNB and ALND (39% vs 28%, p=NS) respectively - Arm pain more common after ALND than SLNB (30% vs 12%, p=0.0045) - LR after ALND: 6% and 0% after SLNB, p=0.0172. - The ipsilateral shoulder flexion restricted in 16 (34%) ALND patients and in 15 (16%) SLNB patients, p=0.0175. |
| Lopez Penha 2011 |  | 145 breast cancer patients (any treatment)  Follow up: >5 years post surgery | Lymphedema – water displacement method, arm circumference | - LR using arm circumference: 23/145 (16%) - SLNB: 24/76 (32%) and ALND: 37/69 (54%) - LR using water displacement method: 11/145 (7.6%) |
| Lucci 2007 | RCT | 891 randomly assigned to SLNB + ALND (n = 445) or SLNB alone (n = 446)  Follow up: 1 year | Lymphedema – arm circumference/volume  Lymphedema (reported subjectively) | - Adverse surgical effects reported in 70% (278 of 399) of patients after SLNB + ALND and 25% (103 of 411) after SLNB alone (p<0.001). - Patients in the SLNB + ALND group had more wound infections (p<0.0016), seromas (p<.0001), and paresthesia (p<.0001) than those in the SLNB-alone group. - The difference between the two lymphedema groups, assessed by arm measurements at 30 days (p=0.36), 6 months (p=0.22), and 1 year (p=0.078) - LR at 1 year (subjective report) were 13% (37 of 288) after SLNB + ALND and 2% (six of 268) after SLNB alone (p<or= 0.0001). |
| Lumachi 2009 | Cohort study | Group A (n=54) SLNB alone; Group B (n=48) SLNB followed by ALND using ultrasound scissors (harmonic scalpel); Group C (n=53), ALND using ultrasound scissors; Group D (n=50) traditional ALND dissection  Follow up: Median 22 months (range 18-28 months) | Lymphedema – arm circumference/volume | - Total LR, 15/205 (7%); SLNB, 2/54 (4%); ALND, 13/151 (9%) |
| Mansel 2006 | RCT | 1031 patients randomized: 515 SLNB, 516 ALND  Follow up: 1, 3, 6, and 12 months after surgery | Lymphedema – Arm volume  FACT-B+4 | - Moderate/severe lymphedema reported more often in ALND than SLNB at 1, 3, 6, and 12 months after surgery (e.g 5% versus 13% at 12 months; all p<0.001) - There was statistically significantly more impairment of shoulder flexion and abduction on the ipsilateral side when comparing ALND to SLNB at 1 month after surgery (p=0.004 and 0.001, respectively). - HRQoL and arm functioning scores were statistically significantly better in the SLNB group throughout (all p≤.003). |
| Martin da Silva 2014 | Cross-sectional | 82 (breast-conserving surgery (quadrantectomy or tumorectomy), and ALND at 3 levels)  Follow up: mean time since surgery was 5.78 years (± 4.60). | FACT-B  QuickDASH survey  ROM (goniometer)  Lymphedema – arm circumference/volume | - LR: 28.04% but did not impair QoL or functional capacity - Deficit of 13.64% in shoulder flexion ROM ipsilateral to surgery, 17.19% in abduction ROM and 25.06% in external rotation ROM - Deficit in shoulder ROM was evidenced on contralateral side, with 4.87% in flexion ROM, 6.39% in abduction ROM and 4.53% in external rotation ROM - The mean functional capacity correlated with HRQoL had a negative impact on physical well-being (p<0.001), family well-being (p= 0.012), emotional well-being (p <0.001), functional well-being (p <0.001), breast subscale (p= 0.18), FACT-B TOI (p<0.001), FACT-G TOTAL (p<0.001). |
| Mathew 2006 | Retrospective review | 312 in Group 1 (SLNB) and 194 in Group 2 (ALND)  Follow up: 2 years after surgery | Lymphedema – arm circumference | - Total LR (31/506, 6%); SLNB (7/312, 2.2%); ALND (24/194, 12.3%) |
| McDuff 2019 | Cohort study | 2171 (1,232 underwent a lumpectomy and 1,034 underwent a mastectomy; 199 patients had no axillary surgery performed, 1520 received a SLNB, and 547 received ALND)  Follow up: 4 years median follow up | Lymphedema – arm circumference/volume | - 5-year cumulative incidence of lymphedema was 13.7% - Significant factors associated with lymphedema on multivariable analysis were high preoperative BMI, ALND and RLNR - Those receiving ALND with RLNR experienced the highest 5-year rate of lymphedema (31.2%), followed by ALND without RLNR (24.6%), followed by SLNB with RLNR (12.2%) |
| McLaughlin 2008 | Prospective observational cohort study | 936 (SLNB alone vs SLNB followed by ALND)  Follow up: Median 5 years after surgery | Lymphedema – arm circumference | - Total LR (86/936, 9%); SLNB (31/600, 5%); SLNB+ALND (55/336, 16%) |
| Monleon 2016 | Cohort study | 112 (44 underwent ALND and 68 SLNB)  Follow up: pre-surgery, and at 1, 6 and 12 months after surgery | Strength (dynamometer) | - Internal rotator strength decreased significantly after ALND in the first month, and did not return to pre-surgery levels after one year of follow-up, with a mean difference of 2.26 kg (p=0.011); no significant loss of strength for patients treated with SLNB - The loss of shoulder ROM was only significant in the first month for the ALND group. - The ALND surgery and having had physical/occupational therapy during follow-up were factors associated with strength loss |
| Nagel 2003 | Cohort study | 106 (mastectomy or breast conserving therapy with complete axillary dissection +/- adjuvant RT)  Follow up:  median follow-up of 14.3 months (range 4- 25 months). | Lymphedema - arm circumference/volume  ROM (goniometer)  Pain (VAS) | - 13 patients (12.6%) had lymphedema - Significant relation between adjuvant radiotherapy and lymphedema (p=0.001, OR 37.2) - 39 out of 106 patients (37%) had reduced ROM - 9 out of 11 who had RT (82%) had a restriction in anteflexion and abduction |
| Nesvold 2008 | Cohort study | 263 (MRM vs BCS + ALND)  Follow up: median of 47 months (range 32-87) post-surgery | Lymphedema – arm circumference/volume  ROM (goniometer) | - 20% lymphedema in MRM group vs 8% in BCS group (p=0.02) - In multivariate analysis, lymphedema was associated with a higher number of metastatic axillary lymph nodes [OR1.14, p=0.02], MRM [OR 2.75, p=0.04] and increasing BMI [OR 1.11, p=0.01] - 24% had a restricted ROM in shoulder flexion in MRM compared to 7% in the BCS group (p=0.01) - Shoulder pain reported by 32% in MRM group and by 12% in BCS group (p=0.001) - Increasing observation time, MRM, and increasing BMI were significantly associated with impaired arm/shoulder function |
| Olson 2008 | Cross-sectional | 1,003 patients with SLNB metastasis had immediate (n=425) or delayed (n=578) complete ALND (part of American College of Surgeons Oncology Group (ACOSOG) trials Z0010 and Z0011).  Follow up: 30 days, 6 months and 1 year after surgery | ROM (goniometer)  Lymphedema – arm circumference/volume | - Patients who received immediate ALND had more axillary paresthesia (51% vs. 35%; p<0.0001) and impaired ROM (49% vs. 36%; p<0.0001) at 30 days than those who received delayed ALND - Impaired ROM observed at 30 days improved significantly at 6 months and 1 year, with no difference noted between groups - Patients who underwent delayed ALND had more lymphedema at 6 months (13% vs 10%; p=not significant) but this difference did not remain at 1 year |
| Ozcinar 2012 | Prospective observational cohort study | 218 patients, patients surgically treated for early-stage breast  Follow up: median 64 months | Lymphedema – arm circumference/volume | - LR at 64 months was 7.3%. (BCS: 11.1%, 4.2% and 0.5%; Mastectomy: 15.0%, 3.2% and 1.4%; SLNB: 8.0%, 1.9% and 0.5%; ALND: 18.0%, 5.3% and 1.4%; RT: 14.7%, 6.3% and 1.4%; without RT: 11.4%, 2.1% and 0.5% at mid-term, late period and late period without mid-term lymphedema respectively) - When mid-term and late term lymphedema were excluded, only 4 patients developed lymphedema at late period, with lymphedema rate of 1.8%. - Factors affecting lymphedema in late period were ALND (p=0.005) and RT(p<0.001) |
| Paim 2008 | Cross-sectional | 96 women treated for breast cancer at an outpatient service (who underwent SLNB or ALND)  Follow up: unknown | ROM (goniometer)  Lymphedema – arm circumference/volume  FACT-B  Pain - Short Form and McGill Pain Questionnaire | - The most common complications were pain (57%), impaired shoulder strength (57%), and fibrosis (54%), impaired shoulder ROM (46%) and lymphedema (17%) - The incidence of impaired shoulder flexion (p= 0.01) and lymphedema (p=0 .002) was higher in ALND group - Winged scapula (8.4%) only occurred in the ALND group - HRQoL was significantly correlated with pain (r=0.53, p=0.000) and impaired shoulder strength in flexion (r=0.4; p= 0.000) and abduction (r=0.5, p=0.000) |
| Paiva 2013 | Cross-sectional | 250 women being treated for breast cancer  Follow up: beyond or within 5 years | Lymphedema – arm circumference/volume | - LR: 112/250 (44.8%) - Shoulder joint mobility, restrictions on abduction movements, internal and external rotation, and anterior shoulder adduction were significantly associated with lymphedema (p<0.001). |
| Peintinger 2003 | Prospective study | 56 SLNB (25 patients receiving the SLNB only (Group I) vs 31 patients who underwent the standard level I and II ALND (Group II) when intraoperative frozen section showed metastatic disease)  Follow up: before surgery (t1), 1 week after discharge (t2) and 9–12 months after surgery (t3). | ROM (goniometer)  McGill Pain  EORTC QLQ-C30 and BR23 | - Arm/shoulder pain reported in 36% of patients after SLNB in comparison to 68% receiving ALND - Numbness reported in 4% of patients in the SLNB group vs 19.3% after ALND - Abduction, flexion and horizontal adduction of the affected arm show significant impairment after ALND |
| Perez 2018 | Observational and cross-sectional | 28 women - undergoing surgery and adjuvant treatment of breast cancer  Follow up: at least 6 months post treatment | Lymphedema – arm circumference/volume  Strength – dynamometer  DASH survey | - When surgery was performed on the non-dominant side, a significant difference (p=0.001) was seen between the affected and unaffected sides - Electromyographic activity of women who had nondominant side surgery showed a significant difference in the flexor muscles of the wrist and fingers (p=0.001) when compared to the unaffected upper limb |
| Rietman 2004 | Retrospective study | 55 patients who underwent a modified radical mastectomy or a segmental mastectomy with ALND  Follow up:  Mean follow-up was 2.7 years | Lymphedema – arm circumference/volume  ROM (goniometer)  Pain (VAS)  Strength (dynamometer) | - The most frequent complications: Pain (60%) and reduction of grip-strength (40%) - The prevalence of impaired ROM and lymphedema was 9–16% and 15% respectively - Radiotherapy and chemotherapy were significant factors in the prediction of impaired ROM |
| Rietman 2006 | Prospective cohort study | 204 patients (SLNB or ALND)  Follow up:  pre-surgery and 2 years after surgery | Pain (VAS)  EORTC QLQ-C30 and QLQBR23 | - Patients experienced significantly less perceived disability in ADL and worsening of HRQoL after SLNB compared with ALND - RT to the axilla predicts an additional decrease in shoulder ROM and increase of arm volume |
| Ronka 2005 | Cohort study | 83 patients – SLNB or ALND +/- adjuvant therapies  Follow up: before surgery and again 2 weeks and 3, 6 and 12 months after surgery | Lymphedema – arm circumference/volume  ROM (goniometer)  Pain (VAS) | - 10% in SLNB group and 22% ALND group had impaired flexion of the ipsilateral shoulder (not significant) at 1 year after surgery - Muscle weakness was reported more often by patients in the ALND group compared to SLNB (42% vs 19%; p= 0.014) at 1 year after surgery - A similar increase in the arm circumference was observed in both patient groups 1 year after surgery (80% vs 86%, p=0.084) - Arm morbidity affected work (p=0.004), hobbies (p=0.003) and daily life in general (p=0.002) more often in the ALND group than in patients who underwent SLNB |
| Roy 2018 | RCT | 1544 patients - sALND restricted to cases with positive SLN biopsy (test arm, n = 774) versus SLN biopsy followed by sALND (control arm, n = 770).  Follow up: up to 5 years | Patient reported pain on arm movement. | - The estimated absolute difference in prevalence between the control and the test arm was 13.9% (95% CI 9.6–18.2%) for pain on arm movement |
| Sagen 2014 | Prospective longitudinal cohort | 391 (ALND and SLNB group)  Follow up: 2.5 years | Lymphedema – arm circumference/volume  Strength – dynamometer  Pain (VAS)  ROM (goniometer) | - More complications after ALND than with SLNB after 2.5 years (p<0.05); arm lymphedema (17% vs 3%), grip strength reduction (12% vs 2%), and shoulder abduction-provoked pain (increase of 6% vs decrease of 50%). - At 2.5 years, the prevalence of women in paid work had fallen significantly from 79 to 66 % (p<0.05) in the ALND group and from 68 to 61 % (p<0.05) in the SLNB group |
| Schrenk 2000 | Cohort study | 35 had ALND of Level I and II and 35 had SLNB  Follow up: Mean follow-up 15.4+/- 6.2 months (range 4 –28 months) | Lymphedema – arm circumference/volume  Pain (VAS)  ROM – Scale 0 to 3 | - Significant increase from pre-op to post op measurement was found in the greatest dimension of the arm (upper arm and forearm) in patients after ALND compared to SLNB (p=0.0001) - Significantly more pain in the operated arm after ALND (p=0.0001) compared to SLNB - 6 patients in the ALND group reported minor but significantly decreased arm mobility (p=0.011) |
| Soares 2014 | Cross-sectional | 200 women with invasive breast cancer who underwent breast-conserving surgery (n = 165) or mastectomy (n = 35) with ALND  Follow up: mean 35 months from surgery | Lymphedema – arm circumference/volume  ROM (goniometer)  Pain (Yes/no) | - Lymphedema was found in 7 (3.5%) of patients. - Paresthesia was observed in 106 (53%) of patients. - Reduced ROM was observed in 48 (24%) of the patients - Pain was reported by 55 (27.5%) of patients |
| Tengrup 2000 | Cohort study | 110 consecutive partial mastectomy and axillary dissection  Follow up: baseline, once a year for up to 5 years after the operation | Lymphedema – arm circumference/volume  ROM (goniometer)  Pain (VAS) | - 21/110 (19%) developed lymphedema, 17/75 (22%) in the radiotherapy group and 4/35 (12%) in the group without radiotherapy - 54/110 (49%) of the patients had reduced shoulder mobility, and of these, 44/75 (57%) were in the radiotherapy group and 10/35 (30%) in the group without radiotherapy - 31% of patients were still perceiving some pain five years after the operation |
| Veronesi 2003 | RCT | 516 patients: SLNB and ALND (axillary dissection group)  or SLNB followed by ALND if positive sentinel node (sentinel node group)  Follow up: at 6 months and 24 months after surgery | Lymphedema – arm circumference  ROM – Mobility scale from 0 to 100  Pain (Questionnaire) | - LR at 24 months: 12/100 (12%) in ALND and 0/100 (0%) in SLNB - Arm mobility <80% at 24 months: 21/100 (21%) in ALND, 0/100 (0%) in SLNB - Sporadic pain at 24 months: 34/100 (34%) in ALND and 7/100 (7%) in SLNB - Continuous pain at 24 months: 5/100 (5%) in ALND and 1/100 (1%) in SLNB |
| Voogd 2003 | Cohort study | 332 ALND  Follow up: Mean time after surgery 4.2 years | Lymphedema – arm circumference  ROM (goniometer) | - 26% had LE or reduced abduction - These patients with LE and reduced abduction had a greater difficulty performing house chores, were more likely to have discontinued hobbies, felt more disabled, and were more likely to have treatment from a physiotherapist. |
| Wernicke 2011 | Cohort study | 265 (lumpectomy and either SLNB or SLNB and ALND  Follow up:  Median follow-up 9.9 years | Lymphedema – arm circumference  ROM (assessed by clinicians) | - Total LR: (45/223, 20%); SLNB (5/108, 5%); SLNB+ALND (40/115, 35%) - Statistically significant decreased range of motion in the ipsilateral shoulder (p< 0.0001) in ALND compared to SLNB |
| Wernicke 2013 | Cohort study | 226 women (lumpectomy, either SLNB or SLNB+ALND)  Follow up: median follow-up 9.9 years | Lymphedema – arm circumference/volume  ROM (goniometer) | - The only chronic complications at 10 years were decreased shoulder ROM 46/111 (41.4%) in SLNB and 92/115 (80.0%) in ALND (p<0.0001), paresthesia 12/111 (10.8%) in SLNB and 39/115 (33.9%) in ALND (p<0.0001), and lymphedema assessed by patients 10/111 (10.0%) in SLNB and 39/115 (33.9%) in ALND (p<0.0001). - Clinicians identified chronic lymphedema in 6/111 (5.4 %) of the SLNB and 21/115 (18.3%) of the ALND cohorts, respectively (p<0.0001). |
| Wetzig 2017 | RCT | 1088 randomized to SNBM with ALND if the sentinel node was positive or RAC preceded by sentinel-node biopsy.  Follow up: at 1, 6, and 12 months, and annually for 5 years | Lymphedema – arm circumference/volume | - Only 28 patients (3.3%) had a corrected increase of arm volume [15% from baseline (RAC 5.0% vs. SNBM 1.7% at 5 years) - Significant predictors of lymphedema were surgery type (RAC vs. SNBM), obesity, diabetes, palpable tumor, and weight gain exceeding 10% of baseline value |
| Wilke 2006 | Prospective cohort study | 5327 patients (SLNB)  Follow up: 30 days and 6 months after surgery | Lymphedema – arm circumference/volume  ROM (goniometer) | - At 6 months, 8.6% of patients experienced axillary paresthesia, 3.8% had restricted ROM, and 6.9% developed proximal upper extremity lymphedema - A decreasing age for axillary paresthesia, increasing BMI, and increasing age for upper extremity lymphedema were all significant predictors of surgical complications at 6 months |
| Woo 2018 | Prospective observational study | 430 reconstructions (223 expander-implant, 44 LD flap, and 163 DIEP flap)  Follow up: 52 months on average (range 24 to 120 months) | ROM (goniometer) | - Expander-implant (OR 2.15, p=0.010) and the LD flap (OR 6.27, p< 0001) were significant risk factors for shoulder morbidity, compared to the DIEP flap - Old age (p=0.041), presence of tumor-positive lymph nodes (p=0.014), and neoadjuvant chemotherapy (p=0.002) were independent risk factors for the development of shoulder morbidity |
| Yuan 2019 | RCT | 558 ARM+ALND (study group) versus 665 standard ALND  Follow up: Pre-surgery, 3 months, 6 months, 12 months, 24 months, 36 months | Lymphedema -arm volume (water displacement) | - For group ARM+ALND: At 3 months, 6 months, 12 months, 24 months and 36 months post-op, the LR was 2.6%, 2.9%, 3.1%, 3.3% and 3.3% respectively - For group standard ALND: At 3 months, 6 months, 12 months, 24 months and 36 months post-op, the LR was 6.9%, 10.8%, 12%, 14% and 15.3% respectively |
| Yue 2015 | RCT | 127 standard ALND (control group) versus 138 ARM+ALND (study group)  Follow up: Pre-surgery, 6 months, and annually for 3 years | Lymphedema – arm circumference/volume | - For control group, LR was 33.07% (median follow up: 20 months) - For study group, LR was 5.93% (median follow up: 20 months) |

RCT, randomized-controlled trial; SLNB, sentinel lymph node biopsy; ALND, axillary lymph node dissection; RLNR, regional lymph node radiation; LR, lymphedema rate; LE, lymphedema; SA, scapula alata; IR, Internal rotator/rotation; ER, external rotator/rotation; HRQOL, health related quality of life; ROM, range of motion; BCS, breast conserving surgery; MRM, modified radical mastectomy; IBR, immediate breast reconstruction; RT, radiotherapy; OL, operated limb; HL, healthy limb; AWS, axillary web syndrome; PM, partial mastectomy; UBF, upper body function; BC, breast cancer; IDDD, Deterioration in Daily Living Activities in Dementia; ASP, arm/shoulder problems; ALN, axillary lymph node; SNBM, sentinel node-based management; RAC, routine axillary clearance; ABPI, Accelerated partial breast irradiation- 30 Gy in five daily fractions; WBI, whole breast irradiation - 50 Gy in 25 fractions, plus a 10 Gy in 5 fractions tumour bed boost; DVH, dose-volume histograms; CPSP, chronic post-surgical pain; UED, upper extremity disability; POD, postoperative day; PPSP, Persistent postsurgical pain; CSS, Constant Shoulder Score; PWB, Physical well-being; CON, closure without flap fixation; FFeS, flap fixation using sutures; FF-G, flap fixation using an adhesive tissue glue; PPBR, pre-pectoral breast reconstruction; IORT, intraoperative radiotherapy; EBRT, external beam whole breast radiotherapy; CF-WBI, conventionally fractionated whole-breast irradiation; HF-WBI, hypofractionated whole-breast irradiation; BCTOS, Breast Cancer Treatment Outcome Scale; BIS, Body Image Scale; EWB, emotional well-being; SWB, social & family well-being; CR, conventional radiotherapy; TT, hypofractionated tomotherapy; TM, total mastectomy; TM-R, immediate reconstruction after total mastectomy; ADM, acellular dermal matrices; NRT, no radiotherapy; BRT, only breast radiotherapy; LD, latissimus dorsi; DIEP, Deep Inferior Epigastric Perforator; ART, breast and axilla radiotherapy; DASH, Disabilities of Arm, Shoulder and Hand Questionnaire; VAS, Visual analogue scale; AROM, active range of motion; PROM, passive range of motion; BMI, body mass index; BIS, bioimpedance spectroscopy; ARM, axillary reverse mapping; Functional Assessment of Cancer Therapy – Breast, FACT-B; 36-Item Short Form Survey, SF-36; European Organization for Research and Treatment of Cancer core quality of life, EORTC QLQ-C30; Breast cancer specific module, QLQ-BR23
