# Supplementary figures and images for: Impact of Axillary Lymph Node Dissection and Sentinel Lymph Node Biopsy on Upper Limb Morbidity in Breast Cancer Patients: A Systematic Review and Meta-Analysis
Source: Ann Surg. 2022 Aug 10;277(4):572–80. doi: 10.1097/SLA.0000000000005671 (PMC9994843; doi:10.1097/SLA.0000000000005671)

**
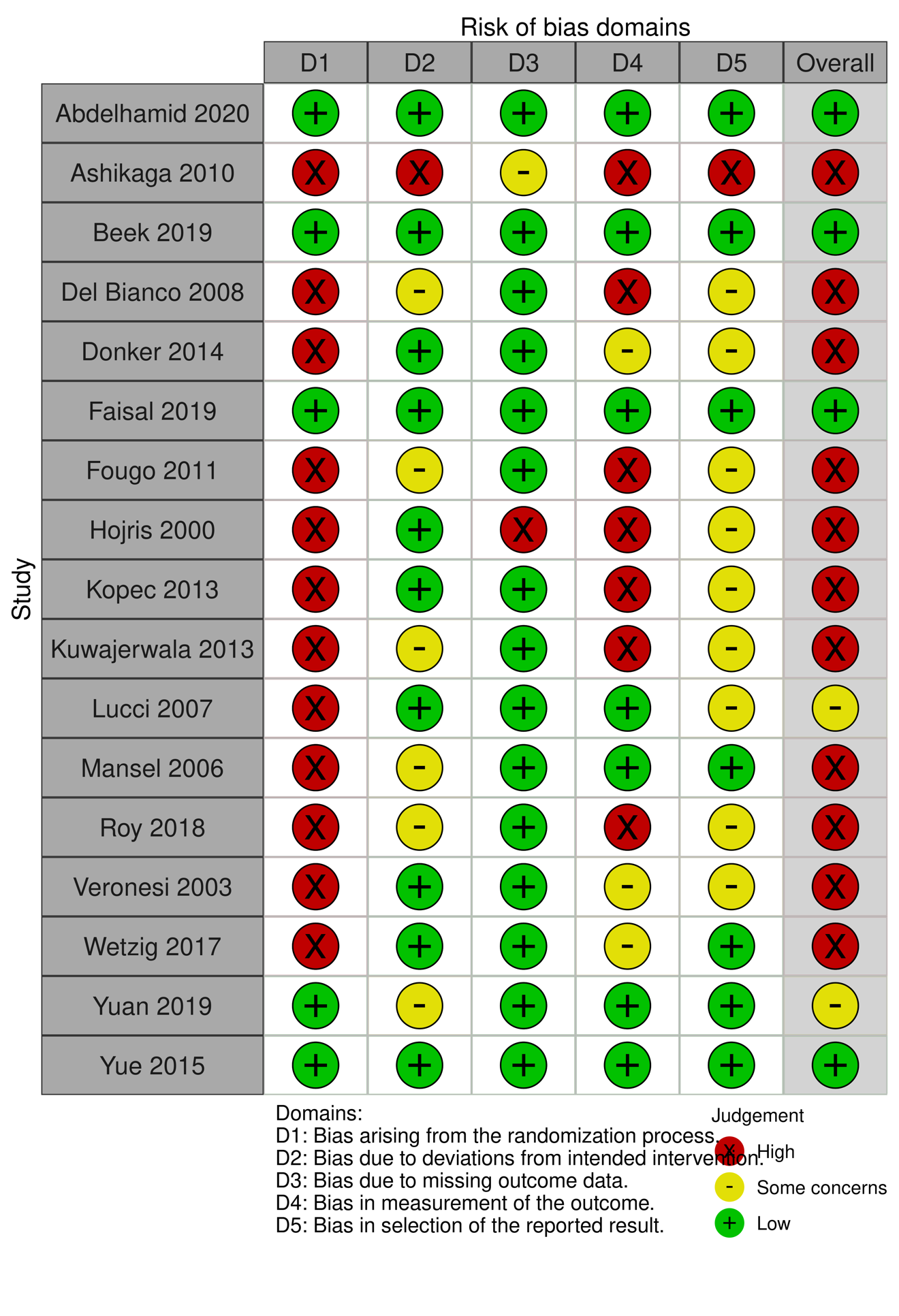
Supplemental Figure 7.** Risk of bias analysis (Rob2) for individual studies (RCTs)

Supplement: Supplementary file 7 [file sla-277-0572-s007.docx]
