# Supplementary material for: Impact of Axillary Lymph Node Dissection and Sentinel Lymph Node Biopsy on Upper Limb Morbidity in Breast Cancer Patients: A Systematic Review and Meta-Analysis
Source: Ann Surg. 2022 Aug 10;277(4):572–80. doi: 10.1097/SLA.0000000000005671 (PMC9994843; doi:10.1097/SLA.0000000000005671)
Supplement: Supplementary file 8 [file sla-277-0572-s008.docx]

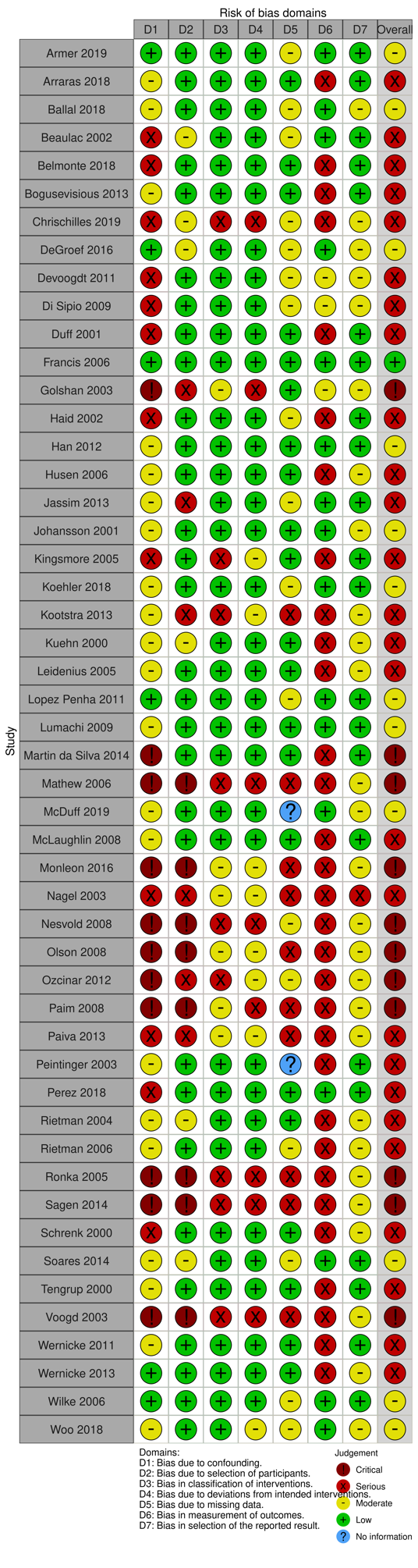
**Supplemental Figure 8.** Risk of bias analysis (ROBIN-I) for individual studies (non-randomized studies)
